# A Sauropod Tooth from the Santonian of Hungary and the European Late Cretaceous ‘Sauropod Hiatus’

**DOI:** 10.1038/s41598-017-03602-2

**Published:** 2017-06-12

**Authors:** Attila Ősi, Zoltán Csiki-Sava, Edina Prondvai

**Affiliations:** 1 Eötvös University, Department of Paleontology, Pázmány Péter sétány 1/C, Budapest, 1117 Hungary; 2 Hungarian Natural History Museum, Baross u 13., Budapest, 1088 Hungary; 3 Department of Geology, Faculty of Geology and Geophysics, University of Bucharest, 1 N. Bălcescu Blvd, 010041 Bucharest, Romania; 4 Department of Biology, Evolutionary Morphology of Vertebrates, Ghent University, K. L. Ledeganckstraat 35, 9000 Gent, Belgium

## Abstract

The lack of sauropod body fossils from the 20 My-long mid-Cenomanian to the late Campanian interval of the Late Cretaceous in Europe is referred to as the ‘sauropod hiatus’, with only a few footprints reported from the Apulian microplate (i.e. the southern part of the European archipelago). Here we describe a single tooth from the Santonian continental beds of Iharkút, Hungary, that represents the first European body fossil evidence of a sauropod from this critical time interval. The mosaic of derived and plesiomorphic features documented by the tooth crown morphology points to a basal titanosauriform affinity suggesting the occurrence of a clade of sauropods in the Upper Cretaceous of Europe that is quite different from the previously known Campano-Maastrichtian titanosaurs. Along with the footprints coming from shallow marine sediments, this tooth further strengthens the view that the extreme rarity of sauropod remains from this period of Europe is the result of sampling bias related to the dominance of coastal over inland sediments, in the latter of which sauropod fossils usually occur. This is also in line with the hypothesis that sauropods preferred inland habitats to swampy environments.

## Introduction

Sauropod dinosaurs were important elements of different Late Cretaceous continental vertebrate communities in Europe. Their record comes, however, mainly from upper Campanian to upper Maastrichtian sediments, and only a very few isolated and fragmentary remains are known from older Upper Cretaceous deposits^1–3^. Almost all of these sporadic remains, both skeletal elements and footprints, have been discovered in Cenomanian localities^4–13^ with some of these even possibly reworked from older, Albian sediments. Accordingly, the late Cenomanian to late Campanian time period, an approximately 20 My long interval^14^, was long thought to represent a hiatus in the European sauropod record^8, 15^. The discovery of some Turonian-Coniacian sauropod footprints in Croatia^1, 16^ and a trackway of a probable small sauropod from the Santonian of Italy^1, 17^, however, seem to challenge this view, and suggest a sampling bias instead^18^, mainly due to the “rarity of inland sediments and dominance of coastal deposits” (Mannion and Upchurch^1^ 2011:529) in the European Upper Cretaceous.

Here we report a sauropod dinosaur tooth from the Santonian of Iharkút, Hungary, an unexpected discovery that represents the first body fossil of the clade known from this poorly sampled period of the sauropod fossil record in the European Cretaceous.

## Material and Methods

The isolated tooth (MTM PAL 2017.1.1.) described here was collected in the Iharkút vertebrate locality (western Hungary) and is housed in the Vertebrate Paleontological Collection of the Hungarian Natural History Museum, Budapest. The specimen was prepared mechanically in the lab of the Hungarian Natural History Museum and the fragmentary margins of the tooth were fixed by cyanoacrylic glue.

The description of the tooth follows the dental terminology proposed by Smith and Dodson^19^. Quantitative shape descriptors such as Slenderness Index (SI: ratio of crown height to maximum mesiodistal width)^20^ and Compression Index (CI: ratio of the maximum labiolingual width to the maximum mesiodistal width of the crown)^2^ were also calculated.

## Locality and geological setting

**Figure 1:**
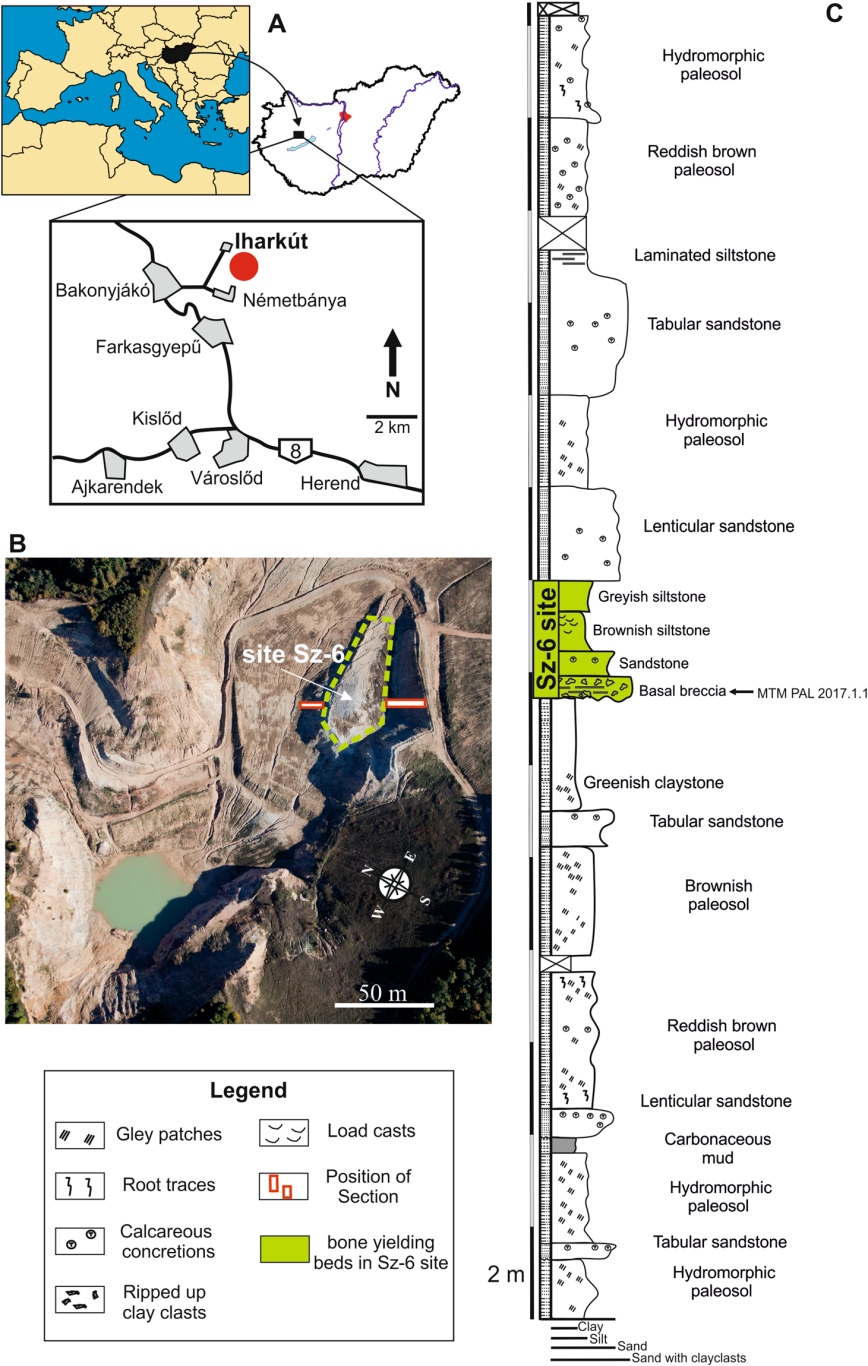
The Santonian Iharkút vertebrate locality (Hungary), and the geological background of site SZ-6. (**A**) Location map of the Iharkút vertebrate locality. (Maps were created by AŐ with Corel Draw 12, http://www.coreldraw.com/en/pages/coreldraw-12/) (**B**), Aerial photo of the Iharkút open-pit, showing the position of site SZ-6. (Photo was taken by Péter Somogyi-Tóth) (**C**), Stratigraphic section of the Csehbánya Formation exposed in the open-pit with site SZ-6 highlighted by green (modified after Botfalvai *et al*.)^21^.

The Iharkút vertebrate locality is in an open-pit bauxite mine near the villages of Németbánya and Bakonyjákó (Bakony Mountains, western Hungary, N47°13′52″, E17°39′01″; Fig. 1A). The oldest rock unit at the locality is the Upper Triassic Main Dolomite Formation, the karstified sinkholes of which were filled up by Cretaceous (pre-Santonian) bauxites (Nagytárkány Bauxite Formation), formerly mined here. The bauxite and the karstified paleosurface is covered by alluvial floodplain deposits of the Santonian Csehbánya Formation consisting of alternating coarse basal breccia, sandstone, siltstone and paleosol beds deposited in a continental environment^21^. Bones at the site are accumulated in bonebeds, among which the most productive one (SZ-6 site, Fig. 1B,C), a greyish, coarse basal breccia layer, produced most of the vertebrate remains including the tooth described in this study. Systematic excavations at the locality resulted in more than 50.000 specimens, represented by isolated and associated bones and teeth of fishes, amphibians, turtles, mosasaurs and other lizards, pterosaurs, crocodyliforms, and dinosaurs, including birds^3, 22^.

## Results

### Crown morphology

**Figure 2:**
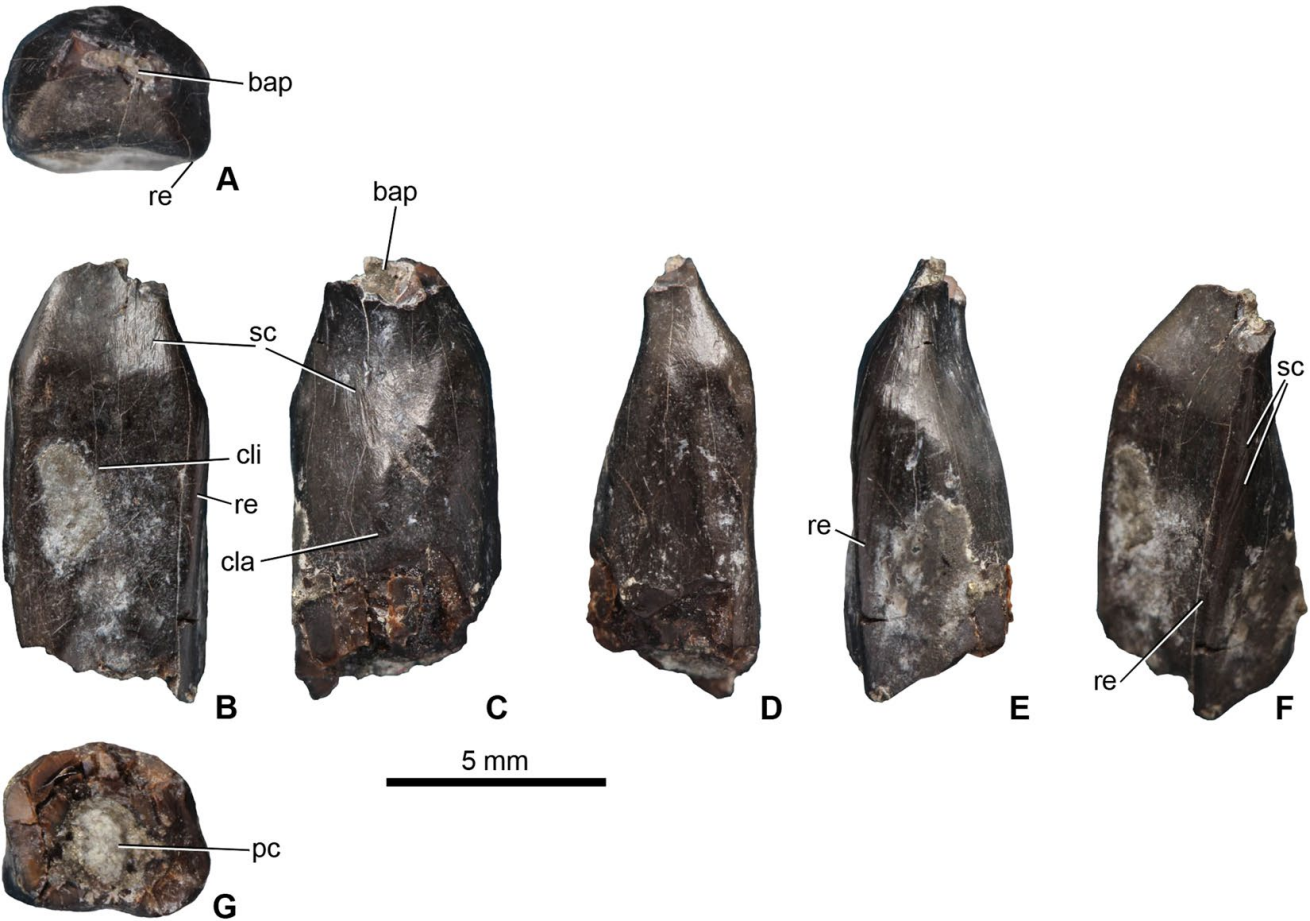
Basal titanosauriform tooth (MTM PAL 2017.1.1.) from the Santonian of Iharkút, Hungary. (**A**) apical, (**B**) lingual, (**C**) labial, (**D**) mesial, (**E**) distal, (**F**) oblique distolingual, and (**G**) basal views. Abbreviations: bap, broken apex of the crown; cla, convex labial surface; cli, slightly concave lingual surface; pc, pulp cavity; re, rounded edge; sc, scratch.

The tooth (MTM PAL 2017.1.1.; Fig. 2) has most of the crown preserved. Apically and basally, however, it is broken, thus the tip and the base of the crown, as well as the root, are missing. The crown is apicobasally elongate (preserved apicobasal height: 10.2 mm) and mesiodistally narrow (4.8 mm) with a minimum SI value of 2.12 (Fig. 2). This gives a minimum log_10_ value of 0.326 for SI that falls just outside of or on the edge of the SI cluster for Macronaria^23^ indicating a relatively wide crown. The mesial and distal margins of the tooth extend parallel to each other before converging apically. Apically, the crown bends labially at first and then seems to incline backwards lingually near its very tip. The lingual surface of the crown (Fig. 2B) does not have a central longitudinal ridge, but is divided into three parts: the basal third is mesiodistally flat with a very shallow depression centrally bordered by shallow, low and rounded mesial and distal buttresses; the central third, albeit placed in the same plane, becomes slightly concave and is still bordered by subtly raised mesial and distal shoulders (‘rounded edge’ in Fig. 2B,E), while the apical third of the lingual surface, gently bending labially, is also slightly concave.

The labial surface is strongly convex (Fig. 2A,D,E), resulting in a D-shaped transverse cross-section at mid-crown, with a CI of 0.79. The same D-shaped cross-section is still present at the base of the crown (Fig. 2G). Apically, the crown becomes more spatulate, labiolingually pinched, than in its basal part. Here, the labial surface also curves mildly labially, mirroring the more marked labial bend of the lingual surface. No distinct grooves or ridges are present on any side of the crown. It is also void of marked carinae, presenting only the two parallel, lingually shifted, low and rounded edges that separate the mesial and distal sides from the lingual surface (Fig. 2B,F). Most of the enamel surface appears to be worn all around the crown; as such, the surface of the crown is smooth and unwrinkled, although covered by feeding-related scratches and pits (see below).

The pulp cavity, filled with pyrite and calcite, can be observed both basally and apically. Whereas its basal section is subcircular in cross-section, apically the pulp cavity becomes strongly labiolingually compressed.

### Tooth wear

**Figure 3:**
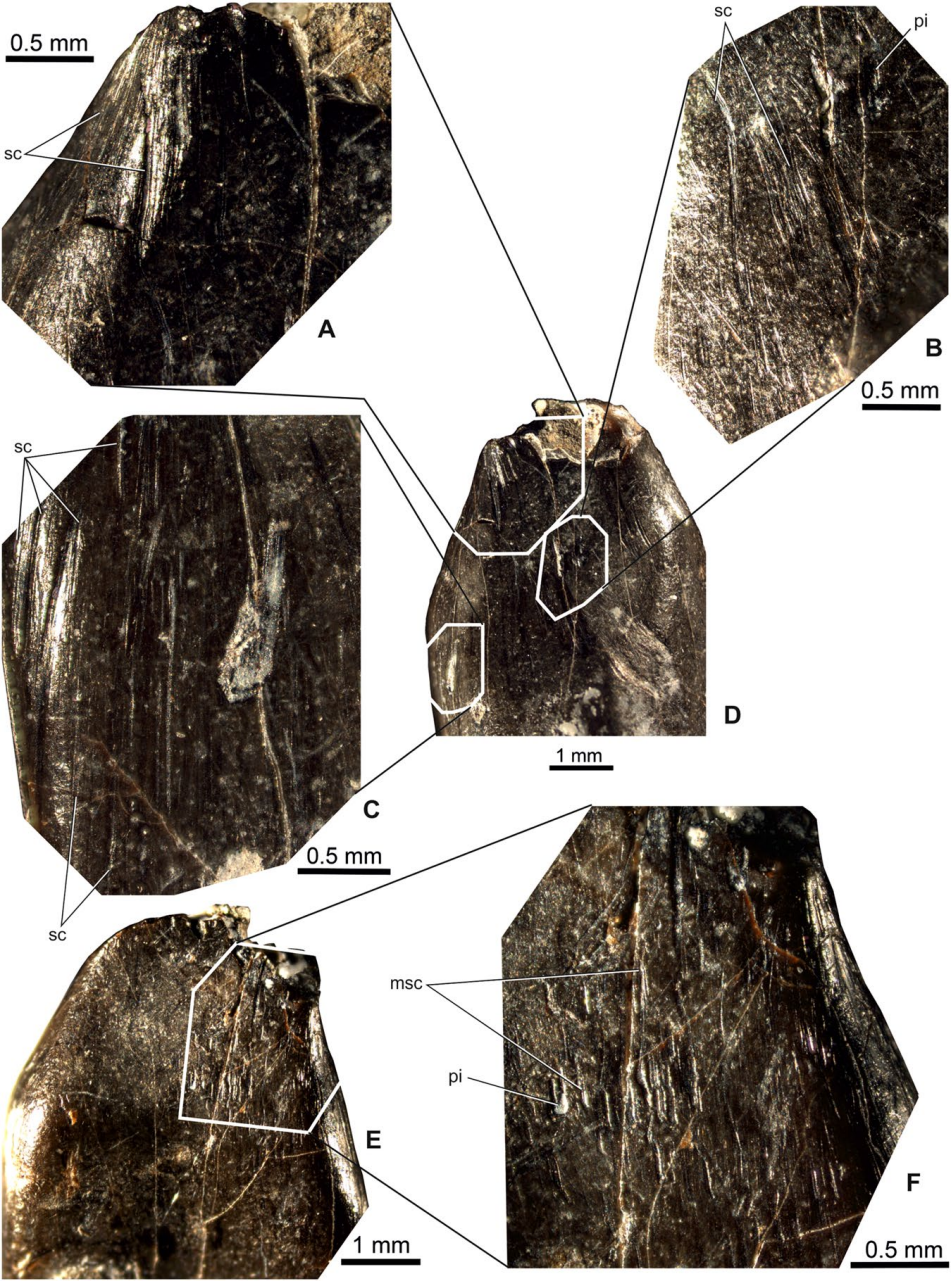
Wear pattern of the basal titanosauriform tooth (MTM PAL 2017.1.1.) from the Santonian of Iharkút, Hungary. (**A–C**) Details of the worn surface of labial (**D**) side. (**E**) Lingual view of the tooth crown; (**F**) ‘meteor shower’ pattern of short scratches and pits on the lingual surface of the crown. Abbreviations: msc, ‘meteor shower’ pattern of short scratches; pi, pit; sc, scratch.

The crown does not show well-distinguished wear facets with exposed dentine, or they may not be preserved due to the missing crown apex (Fig. 3). It seems, nevertheless, that the entire crown was more or less uniformly eroded during life, resulting in hundreds of shorter or longer scratches that are mainly parallel or sub-parallel with the long axis of the crown (Fig. 3A–C). Accordingly, a high orientational consistency is characteristic, with very rare crosswise oriented scratches occurring mainly apically. Scratches are the best developed and longest (over 5–7 mm) along the mesiolabial and distolabial margins of the crown (Fig. 3A,C). Some scratches on the mesial and distal sides are slightly oblique, starting basally from the mesial or distal margin and ending apically on the labial surface. Although scratches are dominant, shallow, apicobasally elongate and triangular pits are also present (Fig. 3F), mainly in the apical third of the crown. A ‘meteor shower’ pattern of short scratches and pits, similar to that reported on the titanosaur teeth from Lo Hueco, Spain^24^, can be observed on the lingual surface of the crown.

Since the tooth crown shows a uniformly eroded pattern, it cannot be ruled out that it is a digested tooth etched by gut acid^25^ resulting in an unwrinkled, enamel-less surface but still leaving the deeper scratches and pits preserved on the dentine surface.

## Discussion

Since this tooth represents the only indication of sauropod dinosaurs in Iharkút up to now, it raises the question whether this specimen might have been reworked from older deposits, as teeth are known to survive relatively long-distance transport and reworking without significant damage^26^. Nonetheless, there are several arguments against this scenario that support the autochthony of the tooth at the site: 1) the tooth has exactly the same dark brownish colour (due to the disperse early diagenetic pyrite present in almost all bones) and general textural pattern as that of the other vertebrate remains from the site; 2) the pulp cavity is lined with a crust of early diagenetic pyrite, in a pattern that is characteristic for many teeth and bones from Iharkút, especially those that have extensive internal cavities, such as theropod and pterosaur bones^27^﻿;3) the tooth is completely void of any signs of abrasion that would have eventually resulted from the interaction between sediment particles and tooth during reworking, 4) the tooth surface is pristine, well-preserved and shows ornamentation as well as features generated only by tooth-food contact. Taken together, these taphonomic features indicate that, similarly to the other teeth and bones preserved in site Sz-6 from Iharkút, the primary depositional setting of MTM PAL 2017.1.1. is represented by the bone-yielding beds of this site.

### Identification and comparisons

Teeth of almost all dentulous vertebrate taxa discovered at Iharkút (from fish to enantiornithine birds) are known from the locality, and MTM PAL 2017.1.1. differs markedly from all of these (see Supplementary information 1), suggesting that it represents a vertebrate taxon not previously identified in the local assemblage. Furthermore, the general shape, morphology and detailed features of the tooth differentiate it from those of most major Late Cretaceous continental vertebrate clades (see Supplementary Information), although it shows remarkable (and somewhat surprising) resemblances to sauropod teeth.

Among sauropods, the tooth MTM PAL 2017.1.1. can be referred to eusauropods based on the possession of a concave lingual surface and a D-shaped crown cross-section^28, 29^. The wrinkled enamel texture characteristic of sauropod teeth^28^ cannot be observed on this tooth, most probably as the result of extensive wear or perhaps of gut acid etching. This condition suggests that the specimen was a functional tooth with prolonged tooth-food contact. However, well distinguished wear facets (such as interlocking V-shaped, high- or low-angled planar facets)^30^ are not present on the preserved part of the crown, making the assessment of tooth-tooth occlusion details impossible. The specimen displays a mosaic of basal and advanced dental features within Eusauropoda. It retains the lingual concavity and a D-shaped cross section, but the tooth crown is narrow and not markedly expanded relative to the root, the labial grooves are absent, and no denticulate mesial and distal margins are present.

The tooth differs from the peg-like teeth of diplodocoids, such as *Diplodocus* ^31, 32^, and the spatulated, mesiodistally wide teeth of non-titanosauriform eusauropods (e.g., *Camarasaurus*)^33^, although the crown curvature in mesial/distal view and the lingual concavity are similar to those seen in *Mamenchisaurus* ^34^. MTM PAL 2017.1.1. is similar to a brachiosaurine tooth from the Lower Cretaceous of Galve, Spain^35^ in having a D-shaped cross-section, concave lingual surface, and parallel, non-carinated mesial and distal margins, although the details of the crown curvature differ slightly. The general form and cross-section of the crown is reminiscent of the premaxillary teeth of the Early Cretaceous North American brachiosaurid *Abydosaurus* ^23^ as well. Some similarities can also be pointed out with the teeth of somphospondylan *Euhelopus* ^36–38^, and those of some indeterminate basal titanosauriforms from the Lower Cretaceous of Japan^39^ that also have parallel-sided crowns with concave lingual surface and relatively low SI values. Nevertheless, they differ from MTM PAL 2017.1.1. in their simple lingual apical curvature, as well as in the presence of a midline ridge within the lingual concavity and of basal lingual buttresses. On the other hand, the tooth markedly differs from the subcylindrical or cylindrical teeth of derived lithostrotian titanosaurs such as *Rapetosaurus* ^40^ or *Nemegtosaurus* ^41, 42^ in having a much lower SI value and a morphologically more complex crown. Indeed, according to the character list of Mannion *et al*.^43^, the Hungarian tooth does not represent a lithostrotian, since it lacks synapomorphies of this clade such as the high-angled planar wear facets (C105) and the cylindrical tooth crown (C109) with a convex lingual surface (C110). The only lithostrotian character present in MTM PAL 2017.1.1. is the absence of an apicobasally orientated lingual ridge (C111).

New discoveries of European latest Cretaceous titanosaurs document an increasing diversity with at least six different taxa (*Ampelosaurus*, *Lirainosaurus, Atsinganosaurus, Lohuecotitan, Magyarosaurus*, and *Paludititan*), among which the first three genera preserve teeth as well^24, 44^, and further isolated, indeterminate titanosaur tooth morphotypes are also reported from different localities from Spain^24^, southern France^2, 45^ and western Romania (pers. observ.). Isolated titanosaur teeth from the Haţeg Basin, Romania, possibly referable to either *Magyarosaurus* or *Paludititan*, are very simple, cylindrical and peg-like, with a mildly convex lingual surface and a high SI value (~5) making these markedly different from the Iharkút tooth. The single known tooth referred to *Ampelosaurus*, and found in a bonebed from southern France^2, 46, 47^, is labiolingually flattened, mesiodistally expanded with mesially and distally positioned longitudinal grooves, again, being clearly distinct from MTM PAL 2017.1.1. Whereas the French taxon *Atsinganosaurus* has gracile, spatulate teeth with a cylindrical crown and mesial and distal ridges extending from the apex to the middle of the crown, the teeth of *Lirainosaurus* from northern Spain are simple cylindrical with a circular cross section^2, 48^ - both of these morphologies are also very different from that of the Iharkút specimen. Besides these three Iberoarmorican taxa, Díez Díaz and colleagues^24^ described two additional morphotypes from the Spanish locality of Lo Hueco. Among them, ‘morphotype B’ is more similar to the Iharkút tooth in having mesiodistally parallel sided crown and shallow ridge-like margins mesially and distally; however, crown curvature and cross section are different. Finally, the ‘Massecaps’ titanosaur tooth morphotype reported by Díez Díaz *et al*.^2^ from southern France and described as ‘robust spatulate’ has a flat lingual surface, without the complex morphology shown by the Iharkút specimen, and lacks the labial bend of the crown in mesial/distal view.

Interestingly, MTM PAL 2017.1.1. bears some resemblance to the isolated and indeterminate sauropod teeth reported from the mid-Lower Cretaceous of western France^49^, especially in the labial bend of the crown at mid-height, followed by a lingual leaning of the tip. Although the teeth figured by Néraudeau *et al*.^49^ are markedly different from the Iharkút specimen in their overall shape, with a more leaf-like contour and asymmetrical, distally deflected apical part, these as well as another unpublished tooth apparently originating from the same site appear to have a similar lingual morphology with a concave basal half flanked by rounded and lingually projecting edges and a more convex apical half. Unfortunately, the affinities of these isolated teeth from western France remain poorly understood, and thus are not useful in shedding light on the affinities of the Hungarian specimen either. Finally, MTM PAL 2017.1.1. is somewhat reminiscent of the dental teeth of the ‘mid’-Cretaceous (Cenomanian-Turonian) basal somphospondylan *Sarmientosaurus* from South America^50^. Although details of the morphology are different, the teeth of *Sarmientosaurus* also show moderate SI values (regarded as intermediate between the broad teeth of basal macronarians and the cylindrical, pencil-like teeth of derived titanosaurs), a D-shaped cross-section of the crown, and more particularly the labially leaning crown at mid-height, below a lingually recurved apical part.

To sum up, specimen MTM PAL 2017.1.1. is certainly a tooth composed of an extensive pulp cavity and dentine covered by heavily worn enamel that shows a number of parallel, elongate scratches along the entire crown. Its morphology, being an elongate non-carinated, spatula-like and pointed tooth, is most closely reminiscent of those of certain sauropods. The mosaic of derived and plesiomorphic characters displayed by the Iharkút tooth clearly suggests a neosauropod affinity. It markedly differs from the peg-like diplodocoid and chisel-like derived titanosaurian teeth (including most titanosaur morphotypes reported previously from the uppermost Cretaceous of Europe), instead being more similar to some brachiosaurid teeth or to those of the basal somphospondylan titanosauriform *Euhelopus* ^38^ and *Sarmientosaurus* ^50^. Thus, we suggest a non-titanosaur titanosauriform affinity for this specimen, pending discovery of further material that might reveal its more precise taxonomic status.

### Status of the European “sauropod hiatus”

Despite being a single piece of evidence, the sauropod tooth from the Santonian of Hungary is of great importance for at least two reasons. First, this specimen is the first sauropod body fossil from a 20 My long hiatus in the fossil record of this clade in Europe, extending from the mid-Cenomanian to the late Campanian interval. Second, the mosaic of derived and plesiomorphic features documented by the crown morphology points to a basal titanosauriform affinity and suggests the occurrence of a clade of sauropods in the Upper Cretaceous of Europe that is markedly different from that encompassing the previously known Campano-Maastrichtian titanosaurs.

Similarly to the ‘sauropod hiatus’ hypothesis proposed by Lucas and Hunt^51^ to account for the absence of sauropod fossils for the largest part of the mid to Late Cretaceous interval in North America, Le Loeuff ^8^ and Le Loeuff and Buffetaut^15^ suggested that the fossil record supports the absence of sauropods from the Cenomanian to late Campanian continental vertebrate record of Europe. This assertion was based on the fact that until the end of the 1990’s not even a single bone or footprint, certainly referable to this group, was known from the, admittedly few, European vertebrate localities representing this time period. The discovery of tracks identified as belonging to small sauropods from the Santonian of southern Italy^17, 52^ and trackways of larger sauropods^16^ (probably titanosaurs)^1^ from the upper Turonian–lower Coniacian of Dalmatia, Croatia, however, indicates that sauropods were present in the Cenomanian to Coniacian continental ecosystems of Europe as well^1, 3^. The sauropod tooth from Iharkút further strengthens this view, filling in the previously hypothesized Late Cretaceous gap in the sauropod fossil record, and shows that instead of their disappearance, the absence of sauropod fossils in European Late Cretaceous assemblages is probably in part the by-product of sampling bias.

Mannion and Upchurch^53^ (2011:534) convincingly demonstrated “the abundance of titanosaurs during the Early and latest Cretaceous and their apparent absence during the mid-Cretaceous” in Europe, and pointed out a positive correlation between the abundance (or lack) of sauropod remains and the amount of terrestrial sediment deposition during the Cretaceous. The Iharkút sauropod tooth came from the deposits of a flash flood event that was formed on a low-lying alluvial floodplain developed not far from swampy/deltaic environments that existed under humid conditions^21^. Accordingly, this landscape was probably more similar to a ‘coastal’ environment than to the much drier and open inland habitats likely preferred by the titanosaur sauropods^29, 53^. The fact that this tooth represents the only fossil of a sauropod discovered so far among more than 50.000 bones and teeth of the Iharkút assemblage fits well into this environmental scenario, but also confirms that sauropods existed in pre-Campanian times within the European archipelago. In addition, the Santonian sauropod fossil evidence from southern Italy and from Iharkút reveals their presence in both the southern^17^ and northern^21^ parts of the Apulian microplate, and suggests their more widespread existence in this region.

The basal titanosauriform affinity of the Iharkút tooth, as assessed based on its mosaic features, might further suggest that the Santonian-aged Iharkút sauropod apparently represented a lineage different from, and more basal than, that of the known European Campano-Maastrichtian sauropods^2, 24, 44, 45, 48, 54, 55^. If this suggested affinity is upheld by future discoveries, the presence of the Iharkút titanosauriform expands the apparently cryptic sauropod diversity in Europe during the Late Cretaceous, from where only lithostrotian titanosaurs^3, 44, 56–58^ have been reported before. It further supports the endemic and relictual nature of these latest Cretaceous European assemblages, highlighted by the presence of a basal titanosauriform sauropod clade that most probably went extinct by Santonian times in most other landmasses^59^.

However, the uncertain taxonomic status of the specimen does not allow a more precise clarification of its affinities and relationships. As such, it also remains unknown whether this form represents an immigrant from Gondwana or Asia, as suggested for some Late Cretaceous European titanosaurs^1, 8, 15^, or it is a relict form that survived in a geographically limited refugium within the European Cretaceous archipelago, a biogeographical phenomenon already pointed out in the case of many other latest Cretaceous continental vertebrates^3, 60, 61^. Certain morphological similarities with the Hauterivian-Barremian aged sauropod teeth from Charentes, western France might support the second scenario, while possible affinities with the ‘mid’-Cretaceous Argentinian *Sarmientosaurus* would rather argue for a southern immigrant. Hopefully further material of the enigmatic Iharkút sauropod will be discovered and will help clarifying this problematic aspect of the Late Cretaceous European biogeography as well.

## Electronic supplementary material

Supplementary_information 1
