# Supplementary material for: A Sauropod Tooth from the Santonian of Hungary and the European Late Cretaceous ‘Sauropod Hiatus’
Source: Sci Rep. 2017 Jun 12;7:3261. doi: 10.1038/s41598-017-03602-2 (PMC5468229; doi:10.1038/s41598-017-03602-2)
Supplement: Supplementary file 1 — Supplementary_information 1 [file 41598_2017_3602_MOESM1_ESM.pdf]

Supplementary information for:

A SAUROPOD TOOTH FROM THE SANTONIAN OF HUNGARY AND THE EUROPEAN LATE CRETACEOUS 'SAUROPOD HIATUS'

by

Attila Ősi, Zoltán Csiki-Sava, Edina Prondvai

Comparison of tooth MTM PAL 2017.1.1. with the teeth of other vertebrates from Iharkút

Teeth of almost all dentulous vertebrate taxa discovered in Iharkút (from fish to enantiornithine birds) are known from the locality, but the tooth MTM PAL 2017.1.1. markedly differs from all of them (see Supplementary figure S1).

Fish show a great diversity in Iharkút with lanceolate teeth in gars (Szabó et al. 2016, Supplementary figure S1A), flattened grinding teeth in pycnodontiforms (Szabó et al. in press, Supplementary figure S1B), and simple, pointed, conical teeth in ellimmichthyform, elopiform, amiiform, and salmoniform fishes (Szabó and Ősi submitted). In addition, fish teeth usually have translucent enamel, unlike that present in MTM PAL 2017.1.1

Teeth of the albanerpetontid amphibians (Szentesi et al. 2013) are very small, simple and chisel-like, being not comparable with MTM PAL 2017.1.1.

Squamates known from the locatity have either conical, curved and carinated teeth with longitudinial enamel ridges (*Pannoniasaurus* Makádi et al. 2012, Supplementary figure S1K) or simple, mono- or multicusped teeth (e.g. *Bicuspidon* Makádi 2006, *Distortodon* Makádi 2013, Supplementary figure S1J) being easily distinguishable from the tooth described herein. In addition, MTM PAL 2017.1.1. is certainly an elongated thecodont tooth which is not the case in these former groups.

Among the thecodont vertebrates, crocodyliform teeth, such as the conical, carinated *Allodaposuchus*-like teeth, the flattened or bulbous, pseudoziphodont *Theriosuchus*-like teeth (Supplementary figure S1C, D), the labiolingually flattened, serrated teeth of *Doratodon* (Rabi and Sebők 2015, Supplementary figure S1E, F), or the anterior low, spatulate and posterior multicusped teeth of *Iharkutosuchus* (Ősi et al. 2007, 2012, Supplementary figure S1G-I) all show different morphologies compared with the tooth MTM PAL 2017.1.1.

Of the theropod dinosaurs, tetanuran, abelisaurid and paravian theropods (including Enantiornithine birds) are known from the locality but their teeth, having typical labiolingually flattened, carinated crowns (Ősi et al. 2010), markedly differ from MTM PAL 2017.1.1 (Supplementary figure S1L, M).

Among ornithischian dinosaurs, ankylosaur teeth are labiolingually compressed with cuspidate mesiodistal edges and labiolingual cingula (Ősi 2005, Supplementary figure S1O). Rhabdodontid and neoceratopsian teeth, being hardly distinguishable from each other, have slightly compressed crown bearing longitudinal enamel ridges both labially and lingually (Virág and Ősi in press, Supplementary figure S1N). Almost all the ornithischian teeth from Iharkút are markedly worn.

It is, nonetheless, reminiscent of sauropod teeth, with its generally straight, cylindrical shape and somewhat widened, spatulated crown.

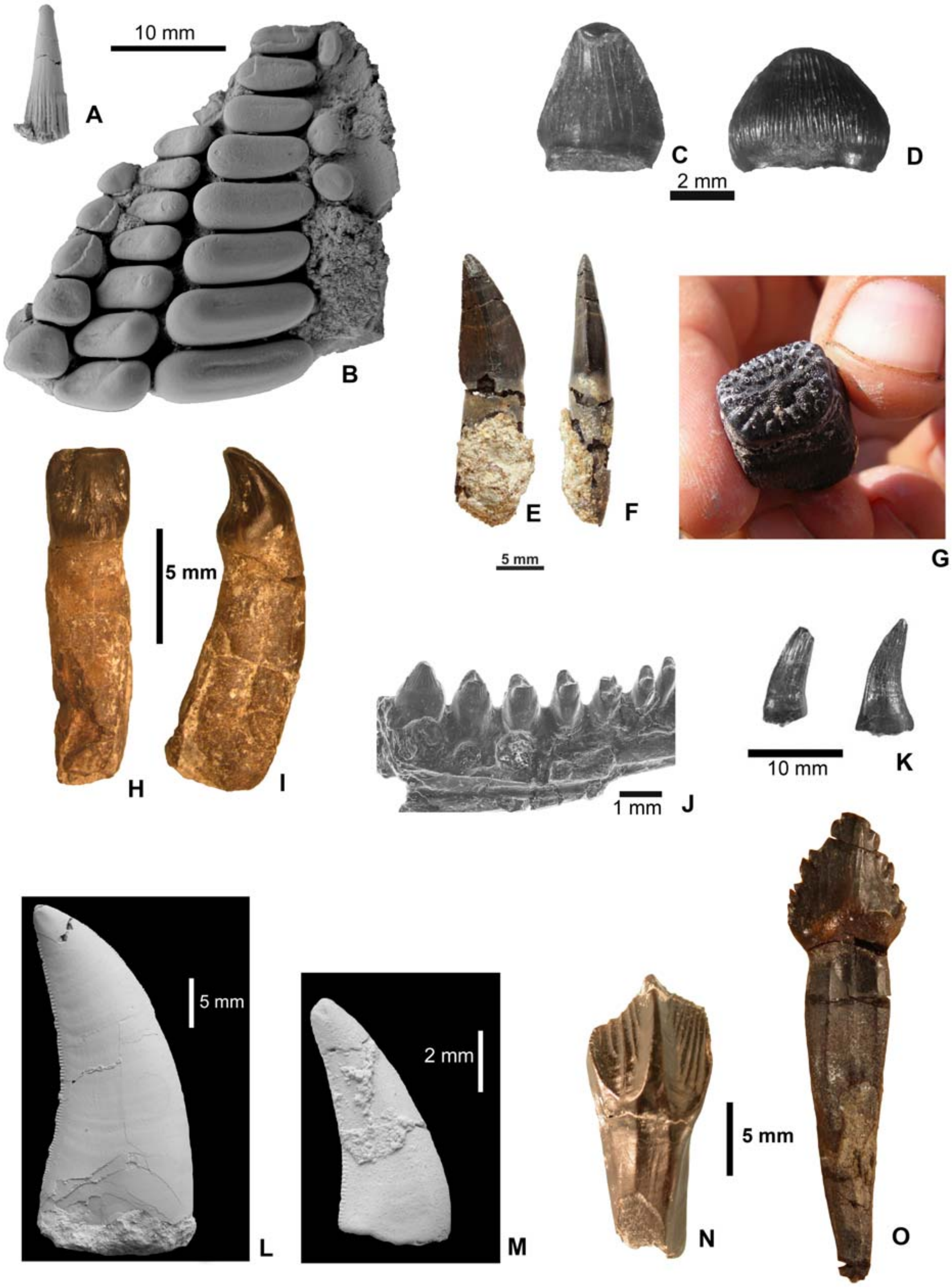

Supplementary figure S1. Teeth of different vertebrates from the Santonian Iharkút vertebrate locality, Hungary. A, Lepisosteiform tooth (modified from Ősi et al. 2012); B, Pycnodontiform jaw element with flat oval teeth (modified from Ősi et al. 2012); C-D,

Mesoeucrocodylia indet. teeth; E, *Doratodon* tooth in lingual/labial view; F, *Doratodon* tooth in mesial/distal view (modified after Rabi and Sebők 2015); G, *Iharkutosuchus* posterior molariform tooth; H, *Iharkutosuchus* anterior spatulated tooth in lingual view; I, *Iharkutosuchus* anterior spatulated tooth in mesial/distal view; J, *Bicuspidon* lower jaw with bicuspid teeth in lingual view (modified from Makádi 2006); K, *Pannoniasaurus* teeth; L, basal Tetanuran tooth; M, Paravian tooth; N, *Mochlodon* tooth; O, Nodosaurid ankylosaur tooth.

References

Makádi, L. *Bicuspidon* aff. *hatzegienzis* (Squamata: Teiidae) from the Upper Cretaceous Csehbánya Formation of Hungary, Bakony Mts. *Acta Geol Hung* **49**(4), pp. 373-385 (2006).

Makadi, L. A new polyglyphanodontine lizard (Squamata: Borioteiioidea) from the Late Cretacoues Iharkut locality (Santonian, Hungary). *Cret Res* **46**:166-176 (2013).

Makádi, L. Caldwell, M. W. & Ősi, A. The First Freshwater Mosasauroid (Upper Cretaceous, Hungary) and a New Clade of Basal Mosasauroids". *PLoS ONE* **7** (12): e51781. doi:10.1371/journal.pone.0051781 (2012).

Ősi, A. *Hungarosaurus tormai*, a new ankylosaur (Dinosauria) from the Upper Cretaceous of Hungary. *J Vert Paleontol*, **25**(2), 370-383 (2005).

Ősi, A., Makádi, L., Rabi, M., Szentesi, Z., Botfalvai, G. & Gulyás, P. The Late Cretaceous continental vertebrate fauna from Iharkút, western Hungary: a review. *Bernissart Dinosaurs and Early Cretaceous Terrestrial Ecosystems* (ed. P. Godefroit), Indiana University Press, pp. 533-568 (2012).

Ősi, A., Apesteguía, S., & Kowalewski, M. Non-avian theropod dinosaurs from the early Late Cretaceous of Central Europe. *Cret Res* **31**:304-320 (2010).

Ősi, A., Clark, J. M., & Weishampel, D. B. First report on a new basal eusuchian crocodyliform with multi-cusped teeth from the Upper Cretaceous (Santonian) of Hungary. *N Jahrb Geol Paleontol*, **243**(2): 169–177 (2007).

Rabi, M. & Sebők, N. A revised Eurogondwana model: Late Cretaceous notosuchian crocodyliforms and other vertebrate taxa suggest the retention of episodic faunal links between Europe and Gondwana during most of the Cretaceous. *Gondw Res*, **28**:1197-1211 (2015).

Szabó, M.,, & Ősi, A. The continental fish fauna of the Late Cretaceous (Santonian) Iharkút locality (Bakony Mountains, Hungary). submitted to *Centr E Geol*.

Szabó, M., Gulyás, P., Ősi, A. Late Cretaceous (Santonian) *Atractosteus* (Actinopterygii, Lepisosteidae) remains from Hungary (Iharkút, Bakony Mountains). *Cret Res* **60**, 239-252 (2016).

Szabó, M., Gulyás, P., Ősi, A. in press. Late Cretaceous (Santonian) pycnodontid (Actinopterygii, Pycnodontidae) remains from the freshwater deposits of the Csehbánya Formation, (Iharkút, Bakony Mountains, Hungary). *Ann Paleontol*.

Szentesi, Z., Gardner, J. D. & Venczel, M. Albanerpetontid amphibians from the Late Cretaceous (Santonian) of Iharkút, Hungary, with remarks on regional differences in Late Cretaceous Laurasian amphibian assemblages. *Can J Earth Sci* **50**(3): 268-281 (2013).

Virág, A., Ősi, A. Morphometry, microstructure and wear pattern of neornithischian dinosaur teeth from the Upper Cretaceous Iharkút locality (Hungary) *Anat Rec*. In press.
